# Mapping Access to Children’s Hospitals in Texas

**DOI:** 10.3390/ijerph21020140

**Published:** 2024-01-26

**Authors:** Shadi Maleki, Nathaniel Dede-Bamfo, Elizabeth Ekren, Milad Mohammadalizadehkorde, Melinda Villagran

**Affiliations:** 1 Translational Health Research Center, Texas State University, 151 Stagecoach Trail, San Marcos, TX 78666, USA; syd14@txstate.edu (E.E.); mvilla@txstate.edu (M.V.); 2 Alkek One, University Libraries, Texas State University, 601 University Drive, San Marcos, TX 78666, USA; nd1115@txstate.edu; 3 Transportation and Public Works Department, City of Austin, 901 South Mopac, Austin, TX 78746, USA; milad.mohammadalizadehkorde@austintexas.gov

**Keywords:** spatial accessibility, children’s hospitals, children’s healthcare, Texas, healthcare access, hospital beds, pediatric emergency, healthcare accessibility, rural health, pediatric healthcare

## Abstract

Vehicle access, travel time, and distance to hospitals and emergency rooms with sufficient patient beds are critical healthcare accessibility measures, especially for children who require specific pediatric services. In a large state like Texas with vast rural areas and limited public transit infrastructure, 75% of the children live over an hour from the closest facility that provides pediatric emergency services or specialty care. In view of this challenge, this study first sought to map the prevailing geographical accessibility to children’s hospitals and, second, to model the hospital beds per capita for each hospital’s service area within the state of Texas. The results showed disparities in accessing emergency pediatric care, especially in rural areas. However, despite major metro areas recording better geographical accessibility to pediatric healthcare, residents in these areas may experience limited hospital bed availability. The findings indicate an urgent need for more pediatric healthcare services in rural Texas. Given the increasing population growth in metro areas and their surroundings, there is also a need for the expansion of healthcare infrastructure in these areas.

## 1. Introduction and Background

In 2015, there were about 30 million pediatric (18-year-olds and younger) emergency visits in the US, out of which 40% were children younger than five [1]. Respiratory and viral infections are among the most common reasons for emergency visits and can sometimes be time-sensitive and potentially life-threatening, especially for children under five [2]. Children’s physiology, anatomy, and medical needs are unique and differ from adults’, thus requiring special treatment and equipment. Meanwhile, many emergency departments do not have the proper equipment and specialized physicians to help pediatric patients [3,4]. A 2022 nationwide surge in cases of respiratory syncytial virus (RSV) highlighted this need for adequate pediatric hospital care, as many hospitals were pushed to capacity [5]. This problem impacted Texas, as more emergency room visits and hospital admissions led to pediatric hospital bed shortages across the state [6]. Although there was a higher volume of patients in urban areas, many Texas rural hospitals’ capacities were pushed to the limit while struggling with personnel shortages, particularly respiratory therapists [7].In addition to viral and infectious diseases, which require immediate access to a hospital with a pediatric emergency unit, chronic diseases such as childhood cancer and newborn complications do not require urgent access to an emergency room, but they do require frequent access to a hospital that provides specialized pediatric care [8]. In both situations, whether a disease requires urgent emergency care or frequent visits, geographic access to a hospital with emergency and specialized pediatric care can impact patients’ chances of receiving care in a timely manner. More generally, long travel distance to healthcare facilities (>60 min) has been identified as a major contributor to under-5 mortality and minimal healthcare utilization [9].

Scholars across many disciplinary perspectives have examined access to healthcare as an essential indicator of human health and well-being [10,11,12]. The US Department of Health and Human Services (USDHHS, Washington, DC, USA) lists access to healthcare as a social determinant of health (SDOH) under the Healthy People 2030 initiative, referring to the quality of an individual’s surrounding environment and social living conditions that affect health and quality-of-life outcomes (USDHHS, Healthy People 2030). The Centers for Disease Control (CDC, Atlanta, GA, USA) and the World Health Organization (WHO, Geneva, Switzerland) also emphasize the importance of equitable access to healthcare within the SDOH framework.

Geographic accessibility is a major dimension of healthcare delivery [13,14]. Geographic or spatial accessibility refers to the ease of physically accessing potential opportunities, and it is often measured based on population distribution (demand), facilities (supply), and transportation [15]. It is common to measure geographic accessibility in terms of driving/walking distance or time [16,17]. Although some studies have not found distance as a determining factor for healthcare usage [18,19], many have found that distance to healthcare facilities can negatively impact patients’ usage of healthcare services [9,20,21,22,23,24]. This phenomenon is known as the distance decay function: the farther away an individual is from services and amenities, the less likely the individual is to use them [25]. Longer travel time to healthcare facilities has also been associated with poorer health outcomes [21], especially for young children in need of immediate care [26].

Access to emergency pediatric care also depends on capacity, commonly measured by available hospital beds per capita [27]. Bed availability is particularly important when there is a greater demand, particularly during a major outbreak or disaster [28]. In the US, pediatric patient units and beds are declining, particularly in rural areas, including rural counties in Texas [29]. Thus, in addition to distance, hospital bed capacity can negatively impact the health of children in need of pediatric acute care in rural areas and urban areas with higher demand rates [30].

Access to a car or reliable alternative transportation is another essential factor influencing healthcare accessibility and better health outcomes in children [31,32]. In an emergency, for example, the availability of a vehicle plays a critical role, especially in rural and remote areas with lower access to different forms of transportation and often at longer distances from a hospital [33]. This is particularly important for people living in rural areas with no cars, as they are more likely to experience a hard time accessing alternative means of transportation [34]. As a result, children living in rural areas often have unmet medical needs [35]. This is even more troubling when children living in rural areas have emergency or frequent specialized health needs that require highly specialized care [36]. In addition, children in rural communities are also more likely to live in socioeconomically disadvantaged communities affected by low income and education levels, putting them at higher risk of not receiving timely and quality medical care [37].

This study examines differences in access to acute care for children who experience critical health issues in Texas. Given the geographic and resource-based elements that reduce healthcare access for many children, the study had two objectives. The first objective was to model and map geographic accessibility to children’s hospitals across Texas (areas within a sixty-minute drive). Children’s hospitals in this paper are defined as hospitals designed exclusively for children or hospitals offering emergency pediatric care. The second objective, on the other hand, focused on modeling capacity (hospital beds per capita) within the sixty-minute drive areas noted above.

## 2. Data and Methods

### 2.1. Study Area

The state of Texas is in the south-central region of the United States, with a population of 29,164,943 [38]. Over 25% of the Texas population is under 18, and about 6.5% (about two million) is under 5 years old [38]. Texas is composed of 254 counties with many major cities, metropolitan areas, towns, and rural areas. Over 83% of its population lives in metro areas such as Austin–San Marcos, Houston–Sugarland, San Antonio–New Braunfels, Dallas–Forth Worth, and El Paso [39].

**Figure 1:**
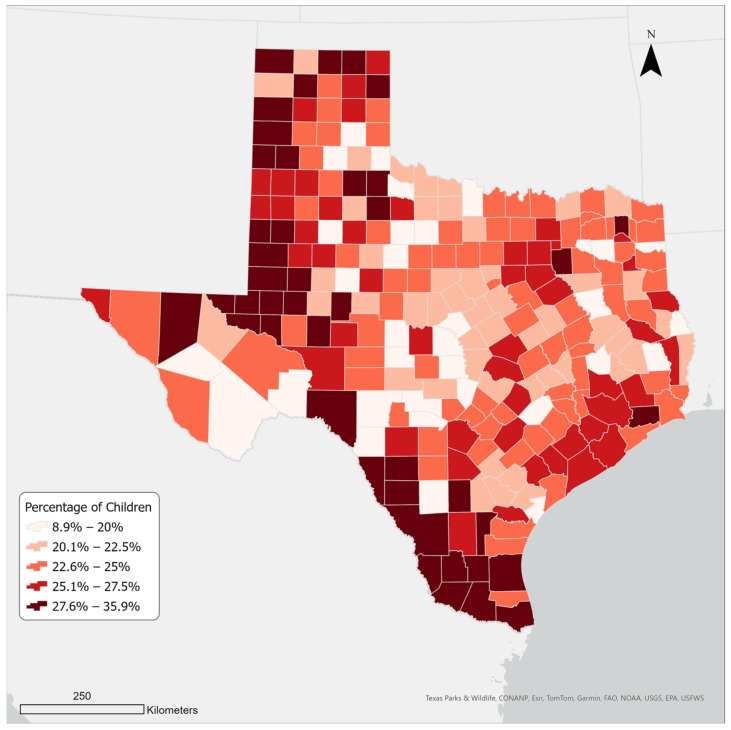
Children’s population density in Texas counties in 2021.

The state’s population has grown steadily over the last two decades. Between 2000 and 2022, Texas gained approximately nine million residents, ranking first in numeric growth among all US states [40]. Domestic migration contributed the most to Texas’s population growth; over 95% of new Texas residents between 2000 and 2022 were people of color [41]. Additionally, the majority of new residents are young, between 20 and 29 [42]. Since 2010, Texas has experienced the highest growth rate in the United States in the under-18 population group [43]. Figure 1 shows children’s (<18 years old) population density in Texas by county in 2021.

### 2.2. Data

A shapefile of US hospitals was obtained from the Homeland Infrastructure Foundation-Level Data (HIFLD). As of November 2022, the dataset contained 16 children’s hospitals in Texas. We also obtained an additional list of children’s hospitals and hospitals with a dedicated pediatric emergency care unit from the Reference Solutions (formerly Reference USA) database accessed through our university. To confirm the validity of the list (whether a hospital was open and had a pediatric care unit offering pediatric emergency care) and the information regarding bed numbers, we checked each hospital’s website and, where necessary, called the hospital for more information. In total, we compiled a list of 24 existing and functional children’s hospitals.

We obtained sociodemographic data on the child population and households without a vehicle through the SimplyAnalytics database accessed by our university. The geographic unit of reference was the county, and the year analyzed was 2021.

### 2.3. Methods and Analysis

#### 2.3.1. Calculating the Sixty-Minute Service Areas

Geographic information systems (GIS) applications are commonly used to identify the shortest driving distance to points of interest. This can be done by using Euclidean distance or by employing road networks. The Euclidean distance straight-line method calculates the shortest distance from one point to another using a straight line, while the network method calculates the shortest distance using road networks. While the difference between distance estimates is not always significant and may not be relevant in non-emergency situations [44,45], using the network distance method is recommended when examining distances in emergency situations, as it provides a more realistic driving time estimate [46]. As a result, we employed network distance in our study.

In relation to the above, a network service area was also adopted as an efficient technique to model geographic accessibility to the hospitals. Unlike the alternatives, mostly potential models, network service areas are widely regarded as a realistic and more meaningful method to measure and visualize accessibility [47,48]. A network service area is defined as “a region that encompasses all streets that can be accessed within a given distance or travel time from one or more facilities” [49]. We utilized Esri’s ArcGIS Online network analyst service to determine 60-min service areas around children’s hospitals. In other words, we determined areas within Texas where residents can readily physically reach a children’s hospital within sixty minutes of traveling by car.

The children’s hospitals were set as the facility locations or origins, while the maximum travel time threshold was set at 60 min. The output layer, which is a polygon feature, was set as an “overlap” rather than a “dissolve”. Although producing visually appealing results, the latter option was considered unsuitable for this research due to the overgeneralization of the service area boundaries. Instead, service areas that uniquely represented each hospital were considered more relevant in this study. In addition, the overlapping service areas provided a more realistic way of estimating beds per capita for children within the service areas. However, overlapping polygons are associated with duplication or double-counting problems. In other words, when overlapping polygons are used as zone features in any spatial operations, they often produce exaggerated results due to their coincident nature. To resolve this spatial problem, we utilized the Count Overlapping Features (COF) tool in ArcGIS Pro 3.1. The COF tool produces a planarized union of the input overlapping areas. In our study, the COF tool resulted in 160 distinct nonoverlapping service areas out of 24 original overlapping service areas.

#### 2.3.2. Calculating Children’s Population within the Sixty-Minute Service Areas

We determined the population of children in each service area based on the population of children at the county level. This included the counties that directly intersected with the service areas. However, to avoid a possible overestimation of the population values, we first determined areas of the counties that are only present in the sixty-minute service areas from the previous step. To obtain such areas, we employed the GIS intersection function. Afterward, we calculated the population of children using the following formula:

*Population of children in a service area = *Σ* (Total County’s population of children ∗ [Intersecting area]/[Total County area])* (1)

#### 2.3.3. Calculating Hospital Beds per Capita within the Sixty-Minute Service Areas

This study used the number of hospital beds as a proxy variable for resource availability, which is considered a primary indicator of healthcare resources [50,51]. To calculate the bed availability within each service area, we used the Generate Near Table tool in ArcGIS Pro 3.1. to find the hospitals within each service area. We joined this table with the service area layer, which contained the population of children for each service area. Then we used the summary statistics tool to calculate the total number of beds within each service area. Finally, we added a new field, divided the number of children by the number of beds, and multiplied it by 1000 to calculate the number of available beds for every thousand children within each service area.

## 3. Results

### 3.1. Geographic Access to Children’s Hospitals

As Table 1 shows, there are 24 children’s hospitals or hospitals with a pediatric emergency care unit that provide children’s healthcare in Texas. These hospitals are located mostly in large metropolitan areas with a large adult population density. While the population of children also tends to be greater in large metro areas, child population density is greater in several rural and micropolitan counties in the southern and north-western regions of Texas (Figure 1).

**Figure 2:**
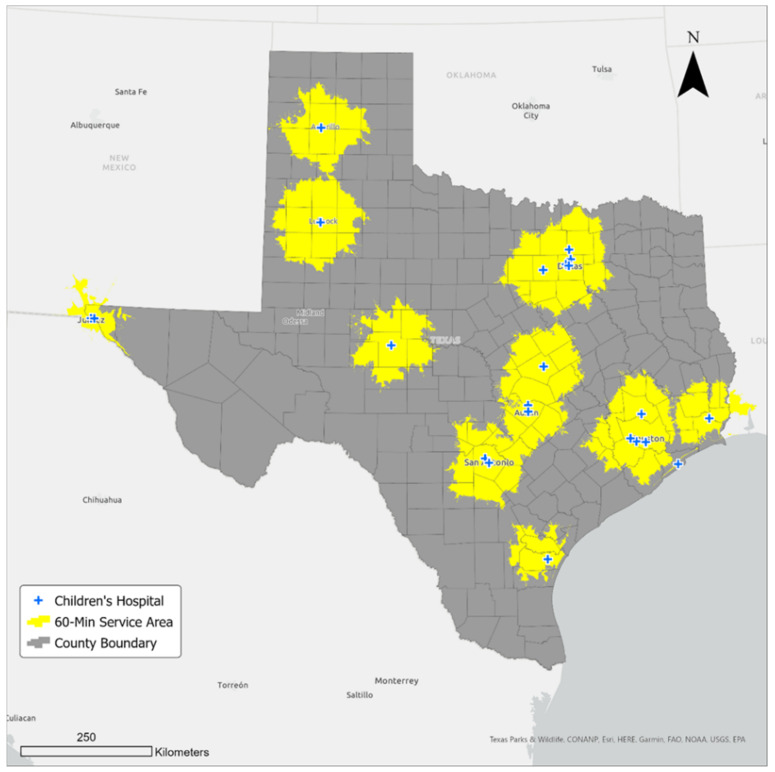
Sixty-minute service areas from children’s hospitals in Texas.

Using network analysis, we modeled a sixty-minute drive service area around each hospital, and we found that the 24 hospitals could serve only 1,852,573 children in 143 counties located within the sixty-minute travel time (Figure 2). Statistics about children’s population and the number of counties served by children’s hospitals in the service area are shown in Table 2. Although most of the 143 counties constitute metropolitan areas, we found a few metropolitan areas in the south (e.g., McAllen–Edinburg Mission metro in Hidalgo County, Laredo metro in Webb County, and Brownsville–Harlingen–Raymondville in Cameron County) with no children’s hospital within a sixty-minute drive. Similarly, the following metro areas in the west and east had no children’s hospitals: Odessa in Ector County, Midland Metro in Martin and Midland counties, Tyler Metro in Smith County, and Texarkana in Bowie County. Overall, over five and a half million children could not access a hospital within an hour’s drive.

**Figure 3:**
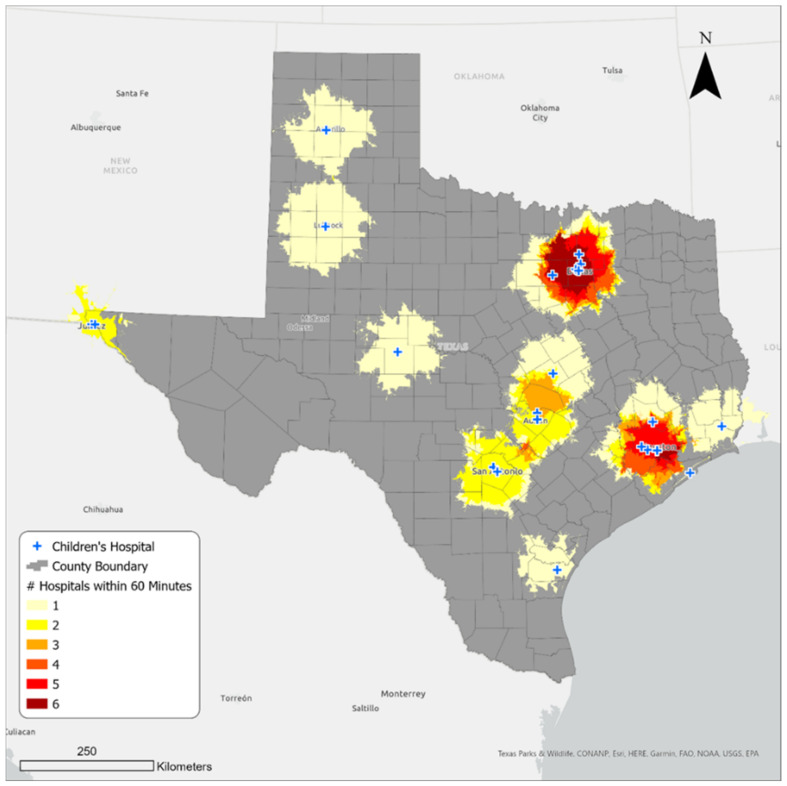
Hospital density in the sixty-minute service areas.

Dallas–Fort Worth and Houston metro areas in the north and southeast of Texas, respectively, have the greatest number of hospitals (Figure 3). Within the areas shaded dark red, at least four hospitals are reachable within a sixty-minute travel time. There are also places in the Austin–Round Rock, San Antonio, and El Paso metropolitan areas that could access more than one children’s hospital within the same travel time. The areas shaded pale yellow have access to one hospital within an hour’s drive.

### 3.2. Bed Availability in Children’s Hospitals in Sixty-Minute Service Areas

**Figure 4:**
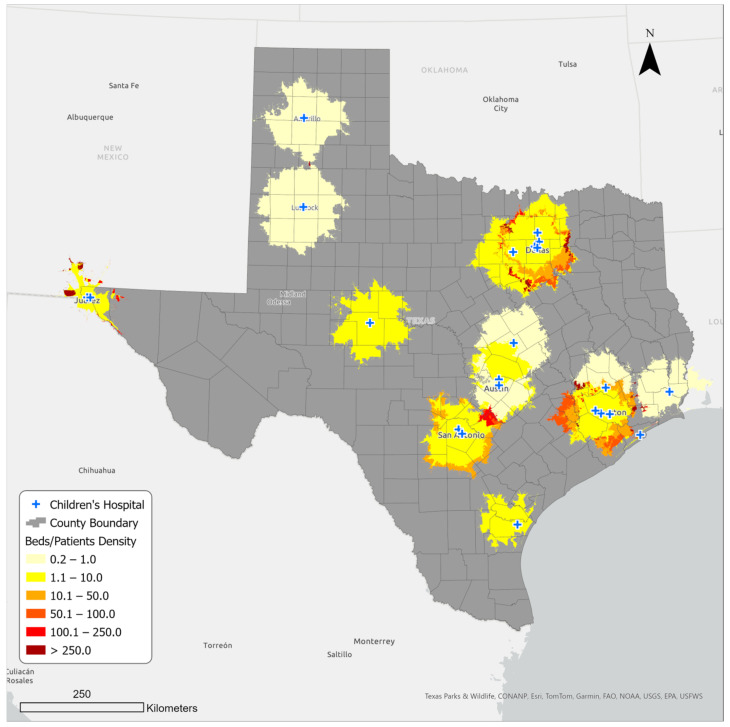
Hospital bed availability in sixty-minute service areas.

Figure 4 presents the beds per capita within the service areas. The beds per capita indicate the potential number of hospital beds for every one thousand pediatric patients within the service areas. Like the hospital density, the bed per capita varies depending on where patients live in the service area. While the dark red areas show over 250 beds available for every one thousand patients, the lighter colored areas show a lower density of up to just 10. Interestingly, the map also reveals that bed availability is not associated with proximity to hospitals. In fact, some areas relatively far from hospitals show greater beds per capita than areas near hospitals. The reason is linked to population density: areas closer to the hospitals are generally metro areas with high child population density and, thus, create a high-demand, low-supply situation. The beds per capita were calculated for each separate overlapping service area and not the entire service area.

### 3.3. Household Vehicle Access in Counties within and outside the Sixty-Minute Service Area of a Children’s Hospital

**Figure 5:**
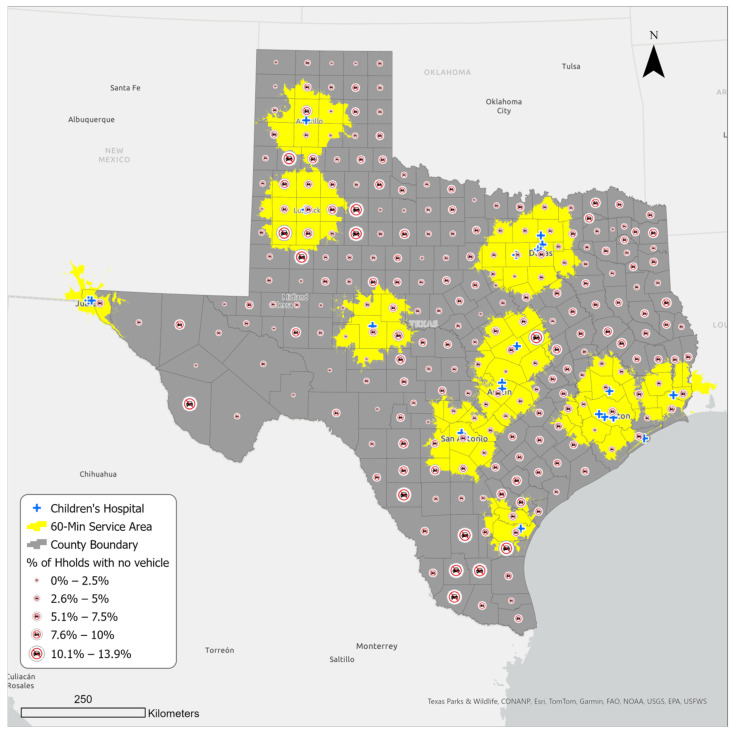
Household vehicle availability in Texas counties and the sixty-minute service areas.

Figure 5 presents household vehicle availability in Texas counties and within the service area. There are close to ten million households in Texas, out of which over seven and a half million (76%) live in the sixty-minute service areas (Table 3). Meanwhile, about 5% of the state household population does not have access to a personal vehicle, and out of this number, 74% live within the service areas (representing about 4% of the overall Texas household population). This means that the remaining 26% (representing 1.4% of the overall Texas household population) do not have a personal vehicle and live outside of the sixty-minute service areas. The latter group thus potentially faces greater challenges in reaching a children’s hospital in case of need; they include counties like Duval, Brooks, Starr, and Jim Hogg in the south; Dickens and Kent in the north; and Presidio in the west. Except for Starr, which is a micropolitan area, the rest of the listed counties are mostly rural and therefore unlikely to have reliable access to public transportation.

## 4. Discussion and Conclusions

This study examined differences in spatial accessibility to acute pediatric care in Texas. The ability to access and receive care at a children’s hospital, or a hospital with specialty expertise to treat children, is widely available to children in more urban areas of Texas, but the same services for the same health issues might not be attainable for children in rural areas where accessibility is limited by a variety of issues.

Spatial accessibility was defined in terms of physical proximity (travel time) to hospitals, the number of hospitals, bed availability, and vehicle availability. In line with past research, we used a sixty-minute travel or drive time as the maximum travel time to model the spatial accessibility to 24 children’s hospitals or hospitals with a pediatric emergency care unit [52,53,54,55]. Geospatial analysis demonstrated that the spatial distribution of hospitals could make them completely inaccessible for children who have limited transportation to access care that is 60 minutes or more away from their home. Although 143 of 254 (56.3%) counties fall completely or partially within the defined service areas that make them reasonably accessible to sick children, this coverage represents only 24% of children across Texas. Additionally, children in 111 rural counties that fall outside the defined healthcare service areas lack access to acute pediatric care when it is needed. In line with previous findings by Chien [30], our study confirms that “the need to drive more than one hour to reach hospital-based pediatric services is more the rule than the exception” (p. 3).

Despite having some of the most renowned children’s hospitals in the United States in 4 of its 10 largest cities, only about a quarter of Texas children live within an hour’s drive of these facilities. The vast majority of children in Texas live 60 min or longer from a children’s hospital, and many of them live in historically medically underserved areas in Texas [56] that lack a facility or provider to make time-sensitive referrals for children who need to be transported by helicopter to major medical centers for care. Even if urban hospitals have airlift capabilities to transport children with acute care needs, if there is no place in a child’s local area to make a referral to a more advanced care facility that could theoretically provide transport to a child with acute care needs, it is unlikely the child could find and access available care elsewhere.

The geographical distribution of rural communities creates significant logistical and staffing challenges to establishing advanced pediatric care facilities; as such, there may be few practical or economic incentives for hospital businesses to establish such facilities in remote communities. This may explain the lack of children’s facilities in these areas. Also, rural residents are more likely to have no health insurance, another potential disincentive for medical businesses to invest in an area [57]. Rural Texas has also historically experienced a high degree of hospital closures [58], suggesting that facilities are difficult to maintain in these areas. Hospital closures impact patients’ access to medical care, especially in emergency and more time-sensitive circumstances, by further reducing already limited options for care [52,59]. Ultimately, the absence of policies and timely interventions to prevent hospital closure and encourage facility development can seriously impact residents’ health, particularly children’s [60]. Public media coverage has also highlighted the importance of timely access to hospitals and medical staff, especially for vulnerable residents [61]. The challenge of timely access to children’s hospitals and specialized medical staff was strongly highlighted at the recent deadliest school shooting in Texas, in Uvalde County, in 2022, when critically injured children had to be transported nearly 80 min to San Antonio, Texas, to receive emergency care [62]. These real-world events support consistent empirical findings that longer travel time to receive medical care affects access [63], which further leads to intensifying disparities in health outcomes across various vulnerable groups of people [64].

This study also found that some metro areas lack a children’s hospital within a sixty-minute drive. This includes areas like McAllen–Edinburg Mission and Laredo metro areas in the south, Midland Metro in the west, and Tyler and Texarkana metro areas in the east. In some of these underserved areas, the population density of children is significantly higher than in many other areas (Figure 1). For example, in a cluster of counties in the south, the population of children alone accounts for over a quarter (27.6%) of the total population. This is also where the two large metro areas of McAllen–Edinburg Mission and Laredo are located. The situation is the same for Ector, Midland, and Martin counties in the west, where the Midland and Odessa metro areas are located. It seems that despite the large share of children, this area is quite marginalized, possibly partly due to its remoteness from other major cities in Texas. This confirms similar findings by Gentili et al. [65], which showed that disparities in accessibility to pediatric healthcare can be a challenge in both rural and urban communities, and other factors present in these areas, such as the insurance type of residents, could impact potential accessibility even further.

In addition, depending on the local population density, bed availability differed within each service area. The lowest density of available beds was found in parts of Austin and Waco areas in central Texas, Lubbock and Amarillo in the northwest, and Woodland and Beaumont in the east. The areas with the best bed availability were those with smaller populations and access to a greater number of hospitals within an hour’s drive. They included areas such as Comal County, located on the San Antonio–Austin corridor in central Texas. The problem of bed availability is highlighted during times of seasonal illnesses such as RSV and other more extreme crises such as the COVID-19 pandemic or the so-called “Tripledemic” in winter 2022, when the co-occurrence of the flu, RSV, and COVID-19 led to widespread hospital bed shortages in Texas [66]. In such situations, bed shortages in emergency rooms and hospitals could cause serious delays in receiving necessary treatments, which can be more threatening to infants and younger children due to their underdeveloped immune systems [67].

Finally, this study examined accessibility in terms of vehicle availability. Although most of the households in Texas have access to a vehicle, a considerable number of households—over half a million—do not. Out of this number, over 135,000 individuals live outside a sixty-minute service area from a children’s hospital, primarily in rural Texas. Some counties in the south, north, and west had a large number of households with no access to a vehicle, and they were also outside of the sixty-minute service area. Thus, children living in these counties were potentially at a higher health risk when emergency care was needed.

Access to transportation is a more complex issue in rural areas [34,63]. In a health emergency, rural residents would most likely not be able to reach a hospital in time unless alternative means of transportation, such as public transportation or helipads, were available [36]. In some cases, even access to better public transportation does not effectively improve healthcare access due to other co-occurring barriers, such as general socioeconomic disadvantage [68]. Also, it is important to consider that the availability of resources such as helipads also does not guarantee access, as many rural residents do not have health insurance or adequate access to information about such available services [37]. Future studies are needed to examine access to transportation and its effectiveness during a pediatric medical emergency.

Geographic location has been found to be an impactful social determinant of health and healthcare access, especially for vulnerable populations, including children [69]. Using GIS, this study was able to analyze pertinent geographic data to examine and visualize spatial access to children’s hospitals and resource availability in terms of available beds per patient at the county level, highlighting the need for immediate action to address the issues regarding access to children’s hospitals in many Texas counties. The results of this study can be used by local officials, state health department and emergency management planning personnel, hospital systems, and medical providers to identify areas lacking care to improve pediatric emergency healthcare accessibility within their local contexts. Since rural children often come to hospital emergency rooms in worse condition than non-rural children because they have not received enough prior care due to distance and availability of care [70], elements of transportation, distance, and travel time as discussed in this study should not be overlooked by policymakers and medical stakeholders. Hospital systems, in particular, should make more robust plans to account for serving these rural populations. Some such hospital-supported interventions have included mobile clinics, drivers or transport services to enhance local EMS capabilities, online scheduling systems that clearly communicate wait and service times to assist in travel decisions, and home-health visits [71]. County and state officials can also support these types of programs through grants and funding, as well as improvements to mobility, travel, and EMS systems that streamline scheduling and medical dispatch efforts more generally. Additionally, local providers like pediatricians and family medicine doctors in rural areas should take note of these findings and recommendations to understand how they can expand their own virtual service offerings and communicate to parents the need to attempt to overcome distance barriers to care before their children’s conditions worsen. Whether at the county, hospital system, or provider level, robust tracking of service utilization and evaluation of distance-related interventions should be tracked in order to understand if these interventions are relevant and being utilized. A lack of improvements to these geographic elements of pediatric care access will result in continued outcomes of reduced health and care utilization indicators in populations of rural children.

Through its findings as well as its limitations, which might be improved upon in other work, this study lays the groundwork for future research examining the impact of geographic location, distance, and resource availability on emergency healthcare access among children. While GIS, as a quantitative tool, is powerful for examining geographic elements of accessibility, it has limitations. For example, GIS can be used to identify locations with no hospitals or far from a hospital, as well as find the closest hospital to a location and provide a comprehensive understanding of spatial patterns. However, future qualitative and place-based research is needed to examine these types of results at a narrower geographic level to identify community-level and other socioeconomic barriers and enablers to pediatric healthcare access that are difficult to measure with only GIS tools. Also, with a focus on acute care, this study only examined spatial accessibility to hospitals that are exclusively dedicated to children’s healthcare and those with an emergency pediatric department. In a future study, the data could be expanded to include other types of healthcare providers, including urgent care and non-pediatric-specific emergency rooms, to provide a more comprehensive view of the accessibility issue.

In addition, accessibility is a multidimensional concept that must be examined through various indicators beyond distance, transportation, and resource (i.e., bed) availability. Other factors such as health literacy, language, racial disparities, access to health insurance, the built environment, perceptions of care quality, perceptions of accessibility, and personal preferences can impact how individuals address and/or overcome these types of geographic barriers in actuality [68,72,73]. Further studies are needed to examine the full effect of these additional factors on pediatric accessibility to better inform stakeholders and policymakers on specific actions to improve various dimensions of access. The current study mainly focuses on spatial accessibility and includes children’s population density and vehicle availability as the only demographic and socioeconomic factors in the analyses. The findings of these additional types of studies could serve to generate recommendations to address issues of access to healthcare among underserved children.

Ultimately, this study intends to be a call to action and a basis for future research that informs interventions and policies to foster health equity.

## Figures and Tables

**Table 1:** Population and household statistics in 2021 in Texas counties.

Characteristic	Value
State population	29,164,943
Children’s population	7,470,055 (25.6% of the total state population)
Average percentage of children per county	24
Number of children’s hospitals	24

Data source: SimplyAnalytics for the demographic data; Homeland Infrastructure Foundation-Level Data (HIFLD) and Reference Solutions for the hospital data.

**Table 2:** Children’s population and number of counties served by children’s hospitals within the sixty-minute drive.

Characteristic	Value
Population of children	7,470,055
Population of children within the sixty-minute drive	1,852,573 (24.8%)
Total number of counties in Texas	254
Texas counties that are completely or partially within the sixty-minute drive	143 (56.3%)

Data source: SimplyAnalytics for the demographic data; the statistics for the service areas were calculated in ArcGIS Pro.

**Table 3:** Vehicle availability and household statistics in Texas.

Characteristic	Value
Number of households in Texas	9,900,865
Number of households in the 60-min service area	7,518,876 (76%)
Number of households outside of the 60-min service area	2,381,989 (24%)
Number of households without a vehicle in Texas	533,500 (5.4%)
Number of households without a vehicle and in the 60-min service area	396,833 (4%)
Number of households without a vehicle and outside the 60-min service area	136,667 (1.4%)

Data source: SimplyAnalytics for the demographic data; the statistics for the service areas were calculated in ArcGIS Pro.

## Data Availability

Data are available on request from the corresponding author.

## References

[1] McDermott K.W., Stocks C., Freeman W.J. (2018). Overview of Pediatric Emergency Department Visits, 2015.

[2] Tregoning J.S., Schwarze J. (2010). Respiratory Viral Infections in Infants: Causes, Clinical Symptoms, Virology, and Immunology. Clin. Microbiol. Rev..

[3] Newgard C.D., Lin A., Malveau S., Cook J.N.B., Smith M., Kuppermann N., Remick K.E., Gausche-Hill M., Goldhaber-Fiebert J., Burd R.S. (2023). Emergency Department Pediatric Readiness and Short-term and Long-term Mortality Among Children Receiving Emergency Care. JAMA Netw. Open.

[4] França U.L., McManus M.L. (2018). Trends in Regionalization of Hospital Care for Common Pediatric Conditions. Pediatrics.

[5] Romo V. (2022). Children’s Hospitals Grapple with a Nationwide Surge in RSV Infections. https://www.npr.org/2022/10/24/1130764314/childrens-hospitals-rsv-surge.

[6] The Texas Tribune (2022). Pediatric Hospitals Short on Beds as Respiratory Illnesses Surge among Children. https://www.texastribune.org/2022/10/28/texas-hospitals-children-flu-covid-rsv/.

[7] My High Plains (2022). COVID, Flu, and RSV Cases Affecting Rural Texas Hospitals. https://www.myhighplains.com/news/local-news/covid-flu-and-rsv-cases-affecting-rural-texas-hospitals/.

[8] Patrick S.W., Davis M.M., Lehmann C.U., O Cooper W. (2015). Increasing incidence and geographic distribution of neonatal abstinence syndrome: United States 2009 to 2012. J. Perinatol..

[9] Quattrochi J.P., Hill K., Salomon J.A., Castro M.C. (2020). The effects of changes in distance to nearest health facility on under-5 mortality and health care utilization in rural Malawi, 1980–1998. BMC Health Serv. Res..

[10] Núñez A., Sreeganga S.D., Ramaprasad A. (2021). Access to Healthcare during COVID-19. Int. J. Environ. Res. Public Health.

[11] Fullman N., Yearwood J., Abay S.M., Abbafati C., Abd-Allah F., Abdela J., Chang H.Y. (2018). Measuring performance on the Healthcare Access and Quality Index for 195 countries and territories and selected subnational locations: A systematic analysis from the Global Burden of Disease Study 2016. Lancet.

[12] Wendt C. (2009). Mapping European healthcare systems: A comparative analysis of financing, service provision and access to healthcare. J. Eur. Soc. Policy.

[13] Maaz M., Papanastasiou A. (2020). Determining the optimal capacity and occupancy rate in a hospital: A theoretical model using queuing theory and marginal cost analysis. Manag. Decis. Econ..

[14] Weiss D.J., Nelson A., Vargas-Ruiz C.A., Gligorić K., Bavadekar S., Gabrilovich E., Bertozzi-Villa A., Rozier J., Gibson H.S., Shekel T. (2020). Global maps of travel time to healthcare facilities. Nat. Med..

[15] Zhao P., Li S., Liu D. (2020). Unequable spatial accessibility to hospitals in developing megacities: New evidence from Beijing. Health Place.

[16] Carrasco-Escobar G., Manrique E., Tello-Lizarraga K., Miranda J.J. (2020). Travel Time to Health Facilities as a Marker of Geographical Accessibility Across Heterogeneous Land Coverage in Peru. Front. Public Health.

[17] Barbieri S., Jorm L. (2019). Travel times to hospitals in Australia. Sci. Data.

[18] Mattson J. (2011). Transportation, Distance, and Health Care Utilization for Older Adults in Rural and Small Urban Areas. Transp. Res. Rec. J. Transp. Res. Board.

[19] Billi J.E., Pai C.-W., Spahlinger D.A. (2007). The effect of distance to primary care physician on health care utilization and disease burden. Health Care Manag. Rev..

[20] Oldenburg C.E., Sié A., Ouattara M., Bountogo M., Boudo V., Kouanda I., Lebas E., Brogdon J.M., Lin Y., for the Étude CHAT Study Group (2021). Distance to primary care facilities and healthcare utilization for preschool children in rural northwestern Burkina Faso: Results from a surveillance cohort. BMC Health Serv. Res..

[21] Kelly C., Hulme C., Farragher T., Clarke G. (2016). Are differences in travel time or distance to healthcare for adults in global north countries associated with an impact on health outcomes? A systematic review. BMJ Open.

[22] Schoeps A., Gabrysch S., Niamba L., Sié A., Becher H. (2011). The Effect of Distance to Health-Care Facilities on Childhood Mortality in Rural Burkina Faso. Am. J. Epidemiol..

[23] Buor D. (2002). Distance as a predominant factor in the utilisation of health services in the Kumasi metropolis, Ghana. GeoJournal.

[24] Goddard M., Smith P. (1998). Equity of Access to Health Care (No. 032cheop).

[25] Pun-Cheng L.S. (2016). Distance decay. International Encyclopedia of Geography: People, the Earth, Environment and Technology: People, the Earth, Environment and Technology.

[26] Rojas-Gualdrón D.F., Caicedo-Velázquez B. (2017). Distancia al centro de atención en salud y mortalidad durante los primeros años de vida: Revisión sistemática y metaanálisis. Rev. Fac. Nac. Salud Pública.

[27] Doi S., Ide H., Takeuchi K., Fujita S., Takabayashi K. (2017). Estimation and Evaluation of Future Demand and Supply of Healthcare Services Based on a Patient Access Area Model. Int. J. Environ. Res. Public Health..

[28] Gausche-Hill M., Ely M., Schmuhl P., Telford R., Remick K.E., Edgerton E.A., Olson L.M. (2015). A National Assessment of Pediatric Readiness of Emergency Departments. JAMA Pediatr..

[29] Cushing A.M., Bucholz E.M., Chien A.T., Rauch D.A., Michelson K.A. (2021). Availability of Pediatric Inpatient Services in the United States. Pediatrics.

[30] Chien A.T., Pandey A., Lu S., Bucholz E.M., Toomey S.L., Cutler D.M., Beaulieu N.D. (2020). Pediatric Hospital Services Within a One-Hour Drive: A National Study. Pediatrics.

[31] Rees C.P., Hawkesworth S., Moore S.E., Dondeh B.L., Unger S.A. (2016). Factors affecting access to healthcare: An observational study of children under 5 years of age presenting to a rural Gambian primary healthcare centre. PLoS ONE.

[32] Tanser F., Gijsbertsen B., Herbst K. (2006). Modelling and understanding primary health care accessibility and utilization in rural South Africa: An exploration using a geographical information system. Soc. Sci. Med..

[33] Shah T.I., Bell S., Wilson K. (2016). Spatial Accessibility to Health Care Services: Identifying under-Serviced Neighbourhoods in Canadian Urban Areas. PLoS ONE.

[34] Blackstock J., Chae K.B., McDonald A., Mauk G.W. (2018). Achieving access to mental health care for school-aged children in rural communities. Rural Educ..

[35] DeVoe J.E., Krois L., Stenger R. (2009). Do children in rural areas still have different access to health care? Results from a state-wide survey of Oregon’s food stamp population. J. Rural Health.

[36] Cancel-Tirado D.I., Feeney S.L., Washburn I.J., Greder K.A., Sano Y. (2018). Health, Well-being, and Health Care Access in Rural Communities. Fam. Community Health.

[37] Greenberg A.J., Haney D., Blake K.D., Moser R.P., Hesse B.W. (2018). Differences in Access to and Use of Electronic Personal Health Information Between Rural and Urban Residents in the United States. J. Rural. Health.

[38] U.S. Census Bureaua (2021). Quick Facts Texas. https://www.census.gov/quickfacts/fact/table/TX/AGE135221.

[39] Kinder Institute for Urban Research After Census Redefines Urban and Rural, Texas Remains Steadfastly Both. https://kinder.rice.edu/urbanedge/census-redefines-urban-rural#:~:text=As%20a%20percentage%20of%20overall,of%20around%2090%25%20or%20more.

[40] U.S. Census Bureaub (2023). Texas Joins California as State with 30-Million-Plus Population. https://www.census.gov/library/stories/2023/03/texas-population-passes-the-30-million-mark-in-2022.html#:~:text=The%20population%20of%20Texas%2C%20the,the%20next%20largest%2Dgaining%20state.

[41] Commission on Community College Finance Working Group, Texas Demographic Center (2021). Updates on Texas’ Demographic Trends and the On-going Research at TDC. https://demographics.texas.gov/Resources/Presentations/OSD/2022/2022_03_07_CommissiononCommunityCollegeFinanceWorkingGroup.pdf.

[42] Federal Reserve Bank of Dallas (2022). Migration to Texas Fills Critical Gaps in Workforce, Human Capital. https://www.dallasfed.org/research/economics/2022/1129.

[43] Texas Comptroller (2020). Young Texans: Demographic Overview. https://comptroller.texas.gov/economy/fiscal-notes/2020/feb/texans.php.

[44] Hua H., Xie H., Tanin E. Is Euclidean distance really that bad with road networks?. Proceedings of the 11th ACM SIGSPATIAL International Workshop on Computational Transportation Science.

[45] Boscoe F.P., Henry K.A., Zdeb M.S. (2012). A Nationwide Comparison of Driving Distance Versus Straight-Line Distance to Hospitals. Prof. Geogr..

[46] Gutiérrez J., García-Palomares J.C. (2008). Distance-Measure Impacts on the Calculation of Transport Service Areas Using GIS. Environ. Plan. B Plan. Des..

[47] Silalahi F.E.S., Hidayat F., Dewi R.S., Purwono N., Oktaviani N. (2020). GIS-based approaches on the accessibility of referral hospital using network analysis and the spatial distribution model of the spreading case of COVID-19 in Jakarta, Indonesia. BMC Health Serv. Res..

[48] Comber A., Brunsdon C., Green E. (2008). Using a GIS-based network analysis to determine urban greenspace accessibility for different ethnic and religious groups. Landsc. Urban Plan..

[49] Environmental Systems Research Institute (Esri) (2023). ArcMap. https://desktop.arcgis.com/en/arcmap/latest/extensions/network-analyst/itemdesc-generate-service-areas.htm#:~:text=A%20network%20service%20area%20is,from%20one%20or%20more%20facilities.

[50] Bauer J., Klingelhöfer D., Maier W., Schwettmann L., Groneberg D.A. (2020). Spatial accessibility of general inpatient care in Germany: An analysis of surgery, internal medicine and neurology. Sci. Rep..

[51] Song Y., Tan Y., Song Y., Wu P., Cheng J.C.P., Kim M.J., Wang X. (2018). Spatial and temporal variations of spatial population accessibility to public hospitals: A case study of rural–urban comparison. GISci. Remote Sens..

[52] McCarthy S., Moore D., Smedley W.A., Crowley B.M., Stephens S.W., Griffin R.L., Tanner L.C., Jansen J.O. (2021). Impact of Rural Hospital Closures on Health-Care Access. J. Surg. Res..

[53] Siegler A.J., Bratcher A., Weiss K.M. (2019). Geographic Access to Preexposure Prophylaxis Clinics Among Men Who Have Sex with Men in the United States. Am. J. Public Health.

[54] Rayburn W.F.M., Richards M.E., Elwell E.C.B. (2012). Drive Times to Hospitals with Perinatal Care in the United States. Obstet. Gynecol..

[55] Branas C.C., MacKenzie E.J., Williams J.C., Schwab C.W., Teter H.M., Flanigan M.C., Blatt A.J., ReVelle C.S. (2005). Access to Trauma Centers in the United States. JAMA.

[56] Texas Health and Human Services (2023). Health Professional Shortage Area (HPSA) Application. https://experience.arcgis.com/experience/323d93aa45fd43e88515cdf65365bf78/page/Page-1/?views=MUA.

[57] Rural Health Information Hub (2023). Rural Health Disparities Overview. https://www.ruralhealthinfo.org/topics/rural-health-disparities.

[58] The Texas Organization of Rural & Community Hospitals (2023). Rural Hospital Closures. https://www.torchnet.org/advocacy--rural-hospital-closure.html.

[59] Miller K.E.M., James H.J., Holmes G.M., Van Houtven C.H. (2020). The effect of rural hospital closures on emergency medical service response and transport times. Health Serv. Res..

[60] Khullar D., Song Z., Chokshi D.A. (2018). Safety-net health systems at risk: Who bears the burden of uncompensated care?. Health Aff. Forefr..

[61] Carver J.L. (2023). Why Health Care Is Still Hard to Access in Rural Towns Near Texas’ Bigger Cities. The Texas Tribune. https://www.texastribune.org/2023/03/16/texas-health-care-deserts/.

[62] Despart Z., Kriel L., Serrano A., Lee J.S., Hernandez A., Cahlan S., Piper I., Garcia U.J. (2022). Uvalde Records Reveal Chaotic Medical Response as Victims Lost Blood. The Washington Post. https://www.washingtonpost.com/investigations/interactive/2022/uvalde-shooting-victims-delayed-response/.

[63] Akinlotan M., Primm K., Khodakarami N., Bolin J., Ferdinand A.O. (2021). Rural-Urban Variations in Travel Burdens for Care: Findings from the 2017 National Household Travel Survey.

[64] Gujral K., Basu A. (2019). Impact of Rural and Urban Hospital Closures on Inpatient Mortality (No. w26182).

[65] Gentili M., Harati P., Serban N., O’Connor J., Swann J. (2018). Quantifying Disparities in Accessibility and Availability of Pediatric Primary Care across Multiple States with Implications for Targeted Interventions. Health Serv. Res..

[66] Furlow B. (2023). Triple-demic overwhelms pediatric units in US hospitals. Lancet Child Adolesc. Health.

[67] Chan M., Meckler G., Doan Q. (2017). Pediatric emergency department overcrowding and adverse patient outcomes. Pediatr. Child Health.

[68] Liu Q., Liu Z., Lin S., Zhao P. (2022). Perceived accessibility and mental health consequences of COVID-19 containment policies. J. Transp. Health.

[69] Edward J., Biddle D.J. (2017). Using Geographic Information Systems (GIS) to Examine Barriers to Healthcare Access for Hispanic and Latino Immigrants in the U.S. South. J. Racial Ethn. Health Dispar..

[70] Peltz A., Wu C.L., White M.L., Wilson K.M., Lorch S.A., Thurm C., Hall M., Berry J.G. (2016). Characteristics of Rural Children Admitted to Pediatric Hospitals. Pediatrics.

[71] Rural Health Information Hub (2023). Transportation to Support Rural Healthcare. https://www.ruralhealthinfo.org/topics/transportation.

[72] Liu Y., Zhong L., Yuan S., van de Klundert J. (2018). Why patients prefer high-level healthcare facilities: A qualitative study using focus groups in rural and urban China. BMJ Glob. Health.

[73] Sobo E.J., Seid M., Gelhard L.R. (2006). Parent-identified barriers to pediatric health care: A process-oriented model. Health Serv. Res..

